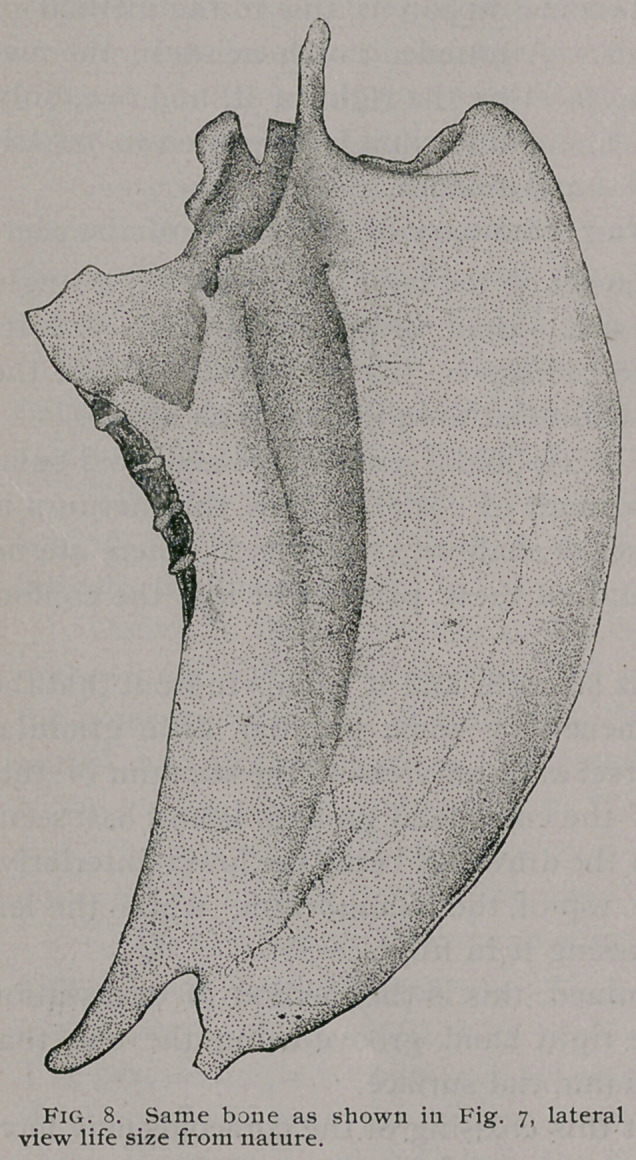# Osteological Studies of the Sub-Family Ardeinæ

**Published:** 1889-07

**Authors:** R.W. Shufeldt


### ART. XV.—OSTEOLOGICAL STUDIES OF THE SUB-FAMILY ARDEINÆ.

BY R. W. SHUFELDT, M.D. C.M.Z.S.

[PART I.]

NOT many years ago, under the name of Herons, ornithological writers and systematists tolerated a very ill-assorted group of birds to constitute a single family, the *Ardeidæ*. Under this head were found associated many alien forms, the majority of which bore some external resemblance to a Heron. Of these we may mention the Storks, now constituting the family *Ciconiidæ*, the Cranes the *Gruidæ*, and many others.

In 1863, Professor Schlegel published a catalogue of Heron-like birds, contained in the Museum of Leyden—in which he admitted sixty-one species and five conspecies, all referred to the genus *Ardea*. This genus he divided into eight sections, to each of which he awarded a name, as the Rail-like Herons, the Egrets and Semi-Egrets, and so on.

Eight years later Dr. G. R. Gray swelled this list to ninety forms—while in 1877, Reichenow recognized but sixty-seven *Ardeidæ*, with a number of sub-species and varieties. This group, the latter writer divided into the three genera, *Ardea*, *Nycticorax*, and *Botaurus*.

The subject of the grouping of the American forms, was first seriously attacked by Mr. Ridgway in his excellent monograph, entitled "Studies of the American Herodiones" the first part of which so far has only appeared.

Here five families of the American Herodiones are presented, viz: the *Cancromidæ*, the *Ardeidæ*, the *Ciconiidæ*, the *Ibididæ*, and the *Plataleidæ*. Sixteen genera of the *Ardeidæ* are given and four species assigned to the genus *Ardea*.

In his introduction to this classification, Mr. Ridgway says: "The water-birds most nearly related structurally to the *Herodiones* are the *Steganopodes*—Pelicans, Cormorants, Gannets, and heir allies—which are likewise both desmognathous and altricial, and what is an important fact in this connection is the circumstance that besides being altricial, they are, with very few exceptions, also decidedly arboreal, most of them even placing their nests on trees. They are swimmers, however, instead of being merely waders."

Ridgway's synopsis appeared in 1878, eleven years after Huxley had published his master-work in avian classification, based upon characters referable to the cranium. In this latter arrangement the Herons fell into the numerous sub-order of the *Desmognathæ*, of which they constituted a part of the group *Pelargomorphæ*. Of this last, the principal osteological characters are said to be that "there are no basipterygoid processes, and the palatines usually unite for a greater or less distance behind the posterior nares; but they send down no vertical plate from their junction."

"The Maxillo-palatines are large and spongy."

"The angle of the mandible is truncated."

"The sternum is broad, and may have two or four posterior notches."

In 1884, appeared the second edition of Professor Coue's key to the North American Birds. This author thoroughly availing himself of the labors of those who had preceded him, says, after fully characterizing the *Order Herodiones*, (Herons and their allies) as *Altricial Grallatores*, including the Herons, Storks, Ibises, Spoonbills, and related birds, that "the group here noted corresponds to the *Pelargomorphæ* of Huxley, the *Ciconiiformes* of Garrod, (minus *Cathartidæ !*) the *Grallatores altinares* of Sundevall, and includes the *Herodiæ*, *Pelargi*, and *Hemiglottides* of Nitzsch, respectively the *Heron* series, the *Stork* series, and the series of *Ibises and Spoonbills*. The first of these differs more from the others than these do from one another. As usual, there are certain out-lying genera, types of families or sub-families, the position of which is not assured. But appearances are that the questionable forms will fall in one or another of the three series indicated. All of these series, to be conventionally rated as sub-orders or super-families are represented in North America. There also all the large and leading families occur."

The *Ardeidæ* of North America have been classified under two sub-families, the *Ardeinæ* containing the true Herons, and the *Botaurinæ*, the Bitterns.

With the latter I shall have but little to do with in the present paper. They have been placed in a single genus under the sub-family just referred to, as constituting the genus *Botaurus*, containing our American Bittern. *B. lentiginosus* and *B. exilis*, the least Bittern (see A.O.U. check-list.) Coues in his last work divides the *Ardeinæ* into the nine genera—*Ardea*, *Herodias*, *Dichromanassa*, *Hydranassa*, *Garzetta*, *Florida*, *Butorides*, *Nyctiardea*, and *Nycterodius*.

The characters upon which a number of these genera rest, seem to me to be scarcely of sufficient importance to warrant such an extensive subdivision. These characters have chiefly been taken from the external, and in several cases trivial differences existing among the forms in question. Such as the matter of dichromatism, some of the Herons having two color phases ; the presence or absence of certain feather ornaments ; and even the comparative lengths of parts have been resorted to, as affording data for such subdivisions.

I am of the opinion that when the structure of the Herons comes to be better known, and is fully taken into consideration, many of these genera will be found to be superfluous. My examinations thus far will not permit me to render a full opinion on this matter, but I have examined the osteology of the *Ardeinæ*, with sufficient thoroughness to convince myself that morphologically the American forms are very closely related. Indeed, I believe the best ends of classification would be met if the present known North American types were restricted to the two genera *Ardea* and *Nyctiardea*, the former to include all the Herons proper, and the latter Night Herons.[1]

My aim in this paper is simply to present a full account of the osteological characters of a typical American Heron, for which purpose I have chosen our well-known and splendid type, *Ardea herodias*, the Great Blue Heron. As we proceed, the comparison with such suitable material, as is on the present occasion available, will in no instance be overlooked, and reference to such books that I may have by me of other anatomists, who have touched upon the structure of the group, will be made, and the results of their investigations fully presented.

---

[1]Since this memoir was written, the Check-List of the American Ornithologists' Union has appeared, (1886), and I see that the Committee who had to do with the classification of Birds, divides the sub-family *Ardeinæ* into the two genera *Ardea* and *Nycticorax*, which practically agrees with my opinions as aboved expressed, and written about a year previous to the appearance of the Check-List. R. W. S.

Garrod did little or nothing with the osteology of the Herons, his researches being confined in them to the study of the condition of the carotids, and certain muscles of the thigh.

*Of the Skull of Ardea herodias:*—Upon superior view of the skull of this Heron, our attention is first directed to its long, narrow, and sharp-pointed bill. This has the outline of a lofty isosceles triangle, of which the base is the line made at the site of the cranio-facial angle, and its apex, the tip of the beak. This surface is pierced in several localities, notably near the apex, and in front of the nostrils, by minute formina, while its sides and ridge are venated. The osseous culmen, owing to a linear depression on either side, passing forwards from the nostril, is in mid-region semicylindrical, which convex surface is continued on the apex, while above the nostrils and the precranio-facial region, though still convex is broader and flatter. The narial apertures are seen from this view, but their true form can best be described from a lateral aspect of the skull.

Across the cranio-facial articulation there is seen a transverse, depressed tract, some three or four millimetres wide, where mesially, the remains of the naso-premaxillary suture is still observable in the adult. This transverse tract is quite thin, and owing to the fact that the ethmoid stops abruptly behind it, beneath, on a line with its posterior boundary, it allows considerable movement in the vertical plane of the bill on the remainder of the skull. How free this is in life I cannot at this moment say, as I have not a Blue Heron in the flesh before me. This depression fixes the boundary very definitely between the frontal and postero-

FIG. 1.

Fig. 1. Superior aspect of the skull of *Ardea herodias*, mandible removed. From a specimen obtained by the author in Wyoming. Same skull used for the figures of that part of the skeleton of this Heron throughout the memoir.

superior region of the upper mandible, and were it not for it, these two surfaces would be continuous, gradually merging into each other, which indeed they almost appear to do now. The frontal region is broad between the superior margins of the orbits, faintly venated, and depressed longitudinally in the middle line.

In the skulls of *Sula bassana* and *Pelecanus fuscus*, specimens of which I have before me, this region is likewise very broad, but the median depression does not exist, it being but faintly marked in the parietal region in these birds.

Upon this upper view of the skull of this Heron, we may also see the superior aspects of the long and large lacrymals. They fit closely to the sides of the frontals, and anteriorly encroach upon the external borders of each nasal.

The posterior orbital margins are pierced by a few minute foramina on either side, into which the larger venations coming from the parietal eminences lead. These latter are quite strongly marked here as they are in other Herons. Among the Pelecans and Gannets, however, this region is not thus distinguished. Still more posteriorly on this aspect we observe the very broad fossa on either side, known as the "crotaphyte fossa." The anterior margin of those fossæ, passes directly across the skull, being simply interrupted in the middle line by a small triangular jog, with its apex directed backwards and continuous with the median line dividing the fossæ. Laterally, these fossæ pass out between the sphenotic and squamosal processes, occupying the entire space. Posteriorly, they are bounded by the supra-occipital line, and a muscular line, on either side, leading to the squamosal process. Fig. 1.

This description of the crotaphyte fossæ of *Ardea herodias*, answers with equal exactness, for the same depressions as four in *Ardea candidissima*, a specimen of which I have before me. In the Yellow-Crowned Night Heron, their form differs materially, (Fig. 29), as well as their position. We observe in this bird that these depressions are separated from each other in the median line, by quite a broad isthmus, which meets the apex of the supra-occipital line. The region below this latter, presenting a prominent, though rounded median crest. In the Night Heron, (*N. violaceus*), too, it can be said, that they are more on a posterior aspect of the skull, rather than on top. This fact is better appreciated by comparing the lateral aspects of the skulls of the two birds. Figs. 1 and 28.

I omitted to point out in passing the difference between the Blue Heron and *Nycticorax violaceus* in so far as the cranio-facial region is concerned, as seen upon this view. Referring to Fig. 29, we will see that the transverse depression described for the Blue Heron is not present in the Yellow-Crowned Heron, the region in the latter being occupied by a shallow concavity. The articulation is quite free, however, in the dried skull, and the relations of the mesethmoid are about the same as in the Blue Heron.

The skeleton of *Nycticorax violaceus* that I am using, is not that of an adult bird—it being "a bird of the year," which I collected at New Orleans, Louisiana, in July, 1883. It probably differs but little, however, from an adult, except in point of size, as the cranial sutures have entirely disappeared. The figures of this Heron illustrating the text below were made from this skeleton, it being, I am sorry to say, the only one in my possession at present.[1]

Upon lateral view of the skull of the Blue Heron, the venated markings of the superior mandible become more evident, and the line leading from the anterior point of the nostril, forwards, is distinctly seen. As we would naturally be led to expect, the inferior and outer border of this mandible is a sharp cutting edge, from the point where it commences by the maxillary, all the way to the apex; and the bill as a whole tapers gradually from base to this latter point.

The outline of the nostril is semi-elliptical, with a broad shelf of bone, extending inwards from its lower margin, and becoming continuous with the general outside surface of the mandible, anteriorly. This shelf does not meet the fellow of the opposite side, as it very nearly does in the Yellow-Crowned Night Heron. Behind, these shelves of bone are directly continuous with the maxillo-palatines. Above them, no nasal septum is present, and an aperture exists to the extent shown in Fig. 2. All traces between the *nasal* and contiguous bones have been absorbed, still

[1]Since writing the above paragraph, Mr. F. A. Lucas has very generously loaned me from his own Cabinet an excellent series of skulls from specimens of *A. egretta*, *A. candidissima*, *A. cærulea*, and *Nycticorax violaceus*, the last mentioned being from an adult individual, and goes to support what I had already recorded throughout this memoir as observed in the skull above referred to in the text. These specimens have been of the greatest assistance to me in the way of verifying the observations made upon the osteology of the *Ardeinæ* upon former occasions.

FIG. 2

Fig. 2. Basil view of the same skull shown in Fig. 1. *mxp*, maxillo-palatine ; *eth*, ethnoid ; *l*, lacrymal. Mandible removed.

such is the conformation of the skull, that one could predict with no little certainty, where their original sites were. Coues states that the "nasal bones are typically holorhinal," (Key. 2d, Ed. p. 647) in the Herons, and so they are, according to the rule laid down by Garrod, for deciding that question, that is, where "a transverse straight line drawn on the skull from the most backward point of the external narial aperture of one side to that of the other, always passes in front of the posterior terminations of the nasal processes of the premaxillæ." (P. Z. S., 1873, p. 35.) This rule, perhaps, will hold better as a guide, than the *form* the nasal bone assumes, for in this Heron there is an evident tendency on the part of its nasal towards schizorhinalism, its posterior narial margin being distinctly angular, at least ; more so even than *Daption capensis*, a skull of which Garrod figured, and one that seems to have a similar tendency. So far as form is concerned, I see the typical holorhinal skull in the Fowl or the *Gallinæ* generally — where the above quoted rule also holds equally good.

Such *single* characters are of great service sometimes, to assist merely in determining a bird's position in the system, but it is hard for me to see how one could think of basing a classification upon such a trivial condition any more than we could upon the shape of the beak itself. Moreover, it would be of little use in such forms as *Sula*, where there is no nostril present, and certainly in some cases violently separates forms that in their general structure closely approach each other.

We find upon lateral view in *Ardea herodias*, a subelliptical aperture, that is bounded anteriorly by the nasal, posteriorly by the lacrymal, and below by the maxillary. Through it can be seen the upper parts of the maxillo-palatines. The *lacrymals* in this Heron are very large bones (Fig. 2); the manner in which one articulates superiorly with the frontal and nasal has already been noted above. Anteriorly the bone has a regularly concave margin, which bounds the aperture alluded to in the preceeding paragraph. Below, a lacrymal rests rather more than its anterior half upon the maxillary, then is slightly raised above it to project backwards as a process with a transversely notched tip. Above this part of the bone there is a constriction which divides it from the larger and upper portion. The surfaces are smooth and the bone is highly pneumatic, air gaining access to its interior through a large foramen on its mesial aspect. Owing to the broad frontals, the orbital roof is very complete, while its outer periphery is sharp and thin. This roof is quite horizontal in *Ardea*, as we see it in the Gannets, but it is inclined to be tilted up in the Night Herons, and consequently not shielding the eye so completely from above.

The *ethmoid* is an unusually thick

FIG. 3.

Fig. 3. Left lateral view of the skull of *Ardea herodias*, same specimen as shown in Figs. 1 and 2. About three-fourths life size.

FIG. 4.

and bulky bone ; it spreads out a wide base for the frontals to rest upon ; its straight anterior upper margin bounds the cranio-facial hinge posteriorly ; its anterior surface is broad, bearing a delicate medial crest, continued upon it from the apex of the rostrum. Its internal structure is cancellous, and air is undoubtedly admitted to permeate it throughout. At a point, on either side, half way between the rostrum and the roof, it supports a feebly developed wing, the lower spur of which meets the backward extending process of the lower and smaller portion of the lacrymal. Among such birds as the Gannets and Pelicans the ethmoid becomes lamelliform again, and it is not nearly as thick, through and through in the Night Herons as we find it in *Ardea*.

The middle third of the rostrum of the Blue Heron is a smooth cylindrical rod, which posteriorly is gradually projected from the sphenoid to merge anteriorly into the ethmoid. The inter-orbital septum is very incomplete, presenting one large vacuity, with which the foramina for the optic nerves have united, together with the smaller nervous foramina found in many birds to the outer side of the latter. Above this interorbital vacuity we note that the olfactory foramen is also very large, and the grooves leading forwards from its anterior apex for the nerve is faintly double. In my specimen of *Ardea candidissima* all these openings have merged into one

Fig. 4. Superior view of the mandible, same specimen as shown in Fig. 3, where the bone is seen on left lateral aspect. Som[illegible]what reduced.

immense aperture, permitting a full view of the interior of the brain-case. This individual is not fully matured, however, and such may not be the case in the adult Egret.[1]

The olfactory foramen of the Yellow-Crowned Night Heron is exceedingly small, while the groove for the passage of the nerve from it, in the specimen before me is single. The optic foramen, likewise, unites with the interorbital vacuity in *Nycticorax*.

The posterior orbital wall in the Blue Heron looks forwards, downwards and slightly outwards; it presents nothing of particular interest; the *foramen ovale* which pierces this wall at its lower part in many birds, has in the Herons moved round so as to appear on direct lateral view, just over the upper edge of the quadrate. Upon this aspect the dome of the parietal eminence is well seen in profile as is the crotaphyte fossa, with the muscular depression behind it.

All Herons present three processes for our examination on the side of the skull, these are, first, the squamosal process seen immediately above and rather in front of the head of the articulated quadrate; second, the sphenotic process defining the boundary of the crotaphyte valley above, and finally, another process just beyond the last, formed at the union of the outer angles of the frontal and squamosal bones.

Sutural traces among the bones composing the infraorbital bar have almost entirely disappeared; they can be made out only after careful scrutiny in my specimen of the Snowy Heron, which, as I have said is not a fully grown bird. In the Blue Heron the line of union between the jugal and quadrato-jugal can sometimes be faintly recognized in the adult individual. This posterior third of the bar is broad and laterally compressed, and the articulation with the quadrate a substantial one, by the usual cup and process joint. The jugal division of the bone is more slender, though also laterally compressed, and the anterior end of the maxillary

---

[1]This does not hold in the skulls of adult specimens of this Heron, for I find since writing the above paragraph, that these foramina in the crania of specimens of *A. candidissima* in the collection of Mr. Lucas as well as in those of this species in the U. S. National Museum, the arrangement is quite like I have described it for *A. herodias*. My thanks are due Mr. Lucas for his courtesy in placing at my disposal the material to which I refer. From the same source I have been enabled to compare skulls of *Botaurus lentiginosus*, *B. exilis*, *Ardea occidentalis*, *A. egretta*, several of *A. candidissima*, *A. virescens*, *Nycticorax*, *N. nævius*, and *N. violaceus*. besides a few skulls and skeletons of Herons from foreign lands. Further on in this memoir I will now introduce some comparisons which this material has enabled me to make.

portion, whose relations are to be described below, is horizontally flattened, though not very broad.

Several years ago Professor Parker found a "post maxillary" in the Emu, and subsequently discovered a similar segment in several of the Herons, as *Ardea garzetta*, *Nycticorax ardeola*, and in the Bitterns, as *Botaurus viridis*, and *B. stellaris*.

This *postmaxillary* is said to be situated or found behind the angle of the maxillary. I find no such bone in the specimens of Herons before me, and can add nothing to the words of Professor Parker given above. It may be that the postmaxillary is present in *Ardea herodias*, but so far absorbed in the adult as not to be recognized, or if a free bone, it has undoubtedly been lost, as my specimens have been in my collection for several years. In either case, fresh material in the flesh, both young and adult, would be required, for me to examine and decide upon such a point.

The *quadrate* of the Blue Heron is a large and massive bone, and indeed, such is its character in all of the *Ardinæ* so far examined by me. Its head presents for our examination two distinct and elongated articular facets, separated by an abrupt and squarish notch. These facets occupy the inner and outer borders of the head of the bone, with their long axis parallel to the long axis of the skull; the outer one, which at the same time is slightly the larger, is in advance of the inner, a circumstance which makes it rather appear that this end of the quadrate was obliquely twisted. Anteriorly, the bone develops a broad lamelliform orbital process, which is flattened behind and convex forwards in front, and gently curved throughout, to the same degree as the posterior wall of the orbit behind it, though it does not touch the latter.

The apex of this orbital process of the quadrate is nicely rounded off, and the anterior surface immediately below its border looks almost directly forwards, a difficult thing to show in a drawing (Figs. 2 and 3).

Posteriorly, the shaft of the quadrate is pierced by a large pneumatic foramen, sufficiently large to permit one to see the trabeculæ spanning its hollow interior, from wall to wall. The massive foot of this bone presents for examination six articular facets. 1. Upon the lateral aspect the usual cup, for the ball and socket joint with the quadrato-jugal. 2. On the extreme outer side of the inferior surface, a sub-elliptical facet, separated from the remaining four by a deep valley. This facet is the largest of the group ; its anterior end is innermost, and it is intended to articulate with a corresponding surface on the mandible. 3. A smaller elliptical facet, with its axis parallel to the last, situated immediately across the valley referred to above. This is the lowest facet of the group, the skull being held with its superior surface, upwards. From the outer side of this facet a *concave* articular surface is carried down, to extend partially across the anterior margin of the intervening valley. 4. A posterior elliptical and smaller facet, higher up on the bone than either 2 or 3 being directed somewhat backwards. A concave narrow articular isthmus connects this facet with No. 2, occupying the posterior margin of the intervening valley. 5. A large circular facet occupying the surface of the inner aspect of the foot of the quadrate, directed downwards, backwards and inwards, the shell being held as above. This facet is separated from 3 by a distinct valley. 6. On the inner angle of the foot of the quadrate, a small circular facet, directed forwards and upwards, intended for the cup on the posterior extremity of the pterygoid. All these articular surfaces except the first and last, have corresponding elevations or depressions for their insertion on the articular end of the mandible, and I have risked the danger of being considered a "dweller upon details" in order to show what an extensive array of facets the foot of this bone supports, and how complicated a surface it offers to the articular extremity of the mandible, I believe that a careful study of these facets, in the class birds, will some day afford us an additional series of facts that can be used with advantage in classification.

The maxillo-palatines, the palatines, the pterygoids, and the condyle of the occiput, can all be seen on direct lateral view, but these I have reserved to describe in the two remaining aspects of the skull.

Seen upon inferior view of the skull the superior mandible presents an unbroken horizontal surface. This is bounded on either side by its sharp edges, while its middle and longitudinal line is defined by a delicate and slightly elevated crest. At irregular intervals on either side of the latter, minute foramina occur, from which spring branching concave venations, directed forwards and outwards to the lateral edges. Fig. 2.

The dentary processes of this premaxillary bone are directed backwards, with pointed apices to overlap the major part of the horizontal plate of each maxillary. Anteriorly the *palatines* merge imperceptibly into the premaxillary, rendering it impossible in the adult Heron to define the exact line of union, their inner margins also uniting with each other, in a like manner, as far back as the middle point on the inferior border of a maxillo-palatine. Here abruptly an interval occurs between them, through which we may see the hinder half of the latter bones and the lower border of the vomer.

Still more posteriorly they become doubly carinated, the posterior angle of the outer keel being bluntly pointed. At the mergence of these keels behind, we find the articular heads for the pterygoids, the upper surfaces of both ride the under side of the rostrum.

Now the inner sides of the inner keels of the palatines are produced forwards to merge into the vomer in a sharp point beyond, thus forming in conjunction with this bone a long doubly carinated process, in the median line, opposite the middle thirds of the palatine bodies. This process forms a part of the lower margin of the vomer, which, as I have said, it really is. The median plate of the vomer rises above this, and extends beyond it, to project slightly into the interspace between the maxillo-palatines. The upper margin of this median vomerine plate is longitudinally split, as it were, and the two thin plates thus formed beautifully curl outwards and downwards, on either side, creating as they do so a median longitudinal groove on the upper aspect of the vomer, over the hinder moiety of which the apex of the rostrum hangs, and even, still more posteriorly, meets it in a free schindylesial articulation. The middle third of the inner border of the upper side of each palatine develops a broad crest that curls outwards all along its summit. On lateral view this crest hides the hinder half of the vomer.

The *maxillo-palatines* of *Ardea herodias* are of a highly spongy bone tissue throughout. This material imperceptibly merges into the more coarsely developed tissue, of a similar character, however, that fills the hollow of the superior mandible beyond. Laterally, the maxillo-palatines may be said to spring from the anterior horizontal plates of the maxillaries, on either side; such a fact is only known to us though from our knowledge of the development of these bones in other birds, for we would hardly suspect it here. The hinder halves of these bones rise parallel to each other, as lofty porous plates, which being produced forwards meet the inner sides of the nasals and the premaxillary to fuse with them. In *Nycticorax* these hinder moieties have a thin outer layer of compact bone tissue covering them which more or less masks their spongy nature.

This Heron has about the same relations existing among the palatines, the vomer, the rostrum of the sphenoid and the bones just described, as we find them in *Ardea*.

In both, too, we find that the median surfaces of the upper part of the inner carination of the posterior third of the palatines is closely applied to each other, so closely in fact that in dried skulls one has to resort to the knife to separate them before we are assured that direct union has not taken place, as we find it in the Pelicans, Gannets and others, where the *entire* median surface of the inner carinal plates fuse to form one descending keel in the middle line.

My specimen of the immature Yellow-crowned Night Heron, shows this union to be of so firm a nature, that it would not surprise me in the least to find in an old adult of this species, that perfect union had taken place between the parts of these inner keels of the palatines that come in contact behind. There is an excellent figure of the base of the posterior half of the skull of *Ardea cinerea* in Professor Huxley's memoir upon the classification of Birds in the Proceedings of the Zoological Society for 1867. Fig. 19, page 437.

It agrees in every detail with the skulls of other true Ardea that I have before me, or have examined elsewhere. A *pterygoid* of the Blue Heron is a straight, stout bone, feebly crested on its upper side, while its inner aspect is grooved for its entire length. Both ends are dilated, the anterior one to receive the palatine head of the corresponding side; the other to articulate with the quadrate. Above this latter extremity a projection is developed on the outer side of which a large pneumatic foramen is seen, which is double in some specimens. This bone is devoid of any sign of a process at the usual site where one usually developes to

Fig. 5. Posterior view of the skull of *Ardea herodias*. Mandible removed. Same specimen as in former figures; life size from nature.

meet the baso-pterygoid apophysis when the latter is present, in birds where this articulation occurs. Other Herons have the pterygoids constructed in precisely the same way. I fail, however, to find the pneumatic foramina in the Night Herons in the same locality as described above for *A. herodias*, but no doubt a larger series of specimens would show it, as the same projection exists in the pterygoid.

In the Blue Heron the basisphenoidal region is elongated and develops a median keel which merges into the inferior surface of the rostrum just beneath the pterygoidal heads. The Eustachian tubes are guarded only by a thin over-arching lamella of bone. As in *Sula* and other forms the basitemporal area is much contracted, while in the dried skull the tympanic cavity is so exposed that no little care is requisite at first to place the parts. The foramina for the pneumagastric, glossopharyngeal, hypoglossal nerves and internal carotid artery, relatively occupy their usual sites, as seen elsewhere in this class.

Upon this inferior view of the skull we really see the under side of the occipital condyle, as its form and articular surface appears only in full view when the skull is looked at directly from behind.

This direct posterior view of a bird's skull is a very instructive one, a fact that was thoroughly appreciated by so talented an anatomist as Garrod, who presented us with a number of them among his valued papers, as for instance where he makes the telling comparisons among the skulls of *Chauna derbiana*, *Chloëphaga magellanica*, and *Mitua tuberosa* (P. Z. S., 1876, pp. 189–200).

Such a view of the skull of this Heron is shown in Figure 5, where the broad crotaphyte fossæ are seen, separated from each other in the median line above by an exceedingly narrow space. The supra-occipital region stands prominently out, partially overhanging the sub-circular foramen magnum. Regularly uniform, with the notch upwards, the large occipital condyle is here better seen, jutting directly backwards from its lower margin. Beneath and in the middle region, the pterygoids and the four carinations of the palatines come into view. These are flanked on either side by the ponderous quadrates, which latter show the large pneumatic foramen in each, leading into their hollow interiors.

Above, on either side, the sphenotic process can be seen, pointing downwards, while below it the squamosal process juts out, and between the two, the crotaphyte fossa passes to the lateral aspect of the skull.

In *Nycticorax* the supraoccipital region is carried to a point above, and is usually divided by a pronounced crest with rounded summit. A far broader strip separates the crotaphyte fossæ from each other in the median line.

The occipital condyle, although of the same shape, is relatively much smaller, and finally the posterior orbital peripheries can be seen peeping above the parietal domes, all these differences enumerated giving to these two skulls, even when only casually compared from this view, a very dissimilar look.

In a number of minor details, principally referable to relative position and form, the points for examination within the brain-case present certain differences between the Night Herons and the genus *Ardea*.

The *mandible* of the Herons offers us a number of points of interest for our investigation.

In the Great Blue Heron (a bird that I have alluded to several times above as simply the Blue Heron) the outer border of either ramas of the mandible for nearly two-thirds of the distance back from the apex is very sharp, and the middle third of the entire length of the bone, the ramal border is swelled just within this cutting edge, which enlargement has its mesial boundary developed also as a sharp border, parallel to, but on a lower plane, with the outer ramal edge.

The inferior ramal borders are rounded for their entire lengths, merging into the gently upward-curved symphysial extremity anteriorly, to be extended behind to the very ends of the articular parts, while on each side they curve towards the median plane. On the external aspect of a ramas we see numerous minute foramina arranged roughly in two longitudinal rows. Some venated markings are also present, No ramal fenestra pierces this bone, where it occurs in many other birds ; but an oblique split plainly marks the site where it was sealed over during the development of the mandibular elements. This entire external surface is smooth and flat, becoming gently convex only as it sweeps beneath the articular ends behind. As I have said, the posterior third of the superior ramal border is somewhat flattened with rounded inner and outer margins. To the rear of the middle of this third, the fairly well developed coranoid processes are seen. They consist of a series of three points in a row, on each side, one behind the other, the anterior being the largest, the other two gradually diminishing in size.

The mesial aspect of either ramas is longitudinally concave for its anterior thirds, while behind, it becomes flattened, to finally pass beneath the articular extremity, facing, as it does so, downwards and towards the median plane.

Viewed from above we find the symphysis concave and more than a sixth the length of the bone. In the median line behind, between the ramal sides, it sends backwards a spike-like process, nearly 2 c. m. long, which we may call the *posterior symphysial process*, this is present in *A. candidissima*, but absent in some specimens of *Nycticorax*. We also see it in very old Albatrosses.

The articular ends above, are generally concave, but two small convexities occurring on the oblique line that crosses in front of the central pit. A circular pneumatic foramen is seen at about where it occurs in the majority of birds where it is present. The hinder ends of these articular extremities are obliquely truncate, (Fig. 5,) the faces looking backwards, upwards, and a little outwards. In the Yellow-Crowned Night Heron these ends are cut square across, and are obliquely concave. In *A. candidissima* they are very much like the Night Herons, though deeper from above downwards, less concave, and face rather more outwardly. Otherwise the mandible of this Heron resembles in every particular the bone as found in the representatives of the genus *Ardea*.

As we might expect, it is built upon the same type also, in *Nycticorax*, differing in no very essential particular. It is proportionately shorter, stouter, and more obtuse ; the ramal vacuity is filled in here also.[1]

There seems to be no exception to the rule that all Herons have the glassohyal of the *hyoid arches* in cartilage, (Fig. 6). Careful examinations made with a good lens fails also in disclosing to me the slightest trace of osseous tissue deposited in the cartilage of the cerato-hyals of adult specimens of *Ardea herodias*. This is equally true of *Garzetta*, *A. candidissima*, but in my immature specimen of *Nycticorax* I find a distinct, though very

---

[1]In closing Part II, of this memoir I will present under "supplementary notes" the cranial comparisons of a number of skulls of Herons, promised on a previous page of the present part.

small osseous cerato-hyal, on either side, embedded in the cartilage of the second visceral arch.

The first basibranchial is compressed from side to side in the *Ardeinæ* generally, with the posterior aspect of the hinder end, fashioned to articulate with the anterior heads of the cerato-branchials, and the head of a slender, styliform second basi-branchial of no great length, which rides above them in the median line. Each cerato-branchial is a long delicate rod of bone, in old individuals often attaining a length of 5.8 c. m., while the epi-branchials rarely exceed a centimetre and a half, are very slight, and have their hinder ends prolonged by needle-like tips of cartilage, a condition which also obtains with the end of the second basibranchial or urohyal.

A specimen of the Snowy Egret before me has the bony parts of the ear so well preserved that I am enabled to see the elliptical stapedial plate, and the delicate bony rod of the mediostapedial part of the apparatus. The sclerotal plates of the eye are elongated and rather narrow, they average from thirteen to sixteen in *Ardea herodias.*

Before entering upon the remainder of the axial skeleton, I will take this opportunity to further say that the tracheal rings also ossify as in other birds. Comparatively, the tube seems to be of small calibre, and I think one would rather be led to look for a larger wind-pipe in so big a bird.

*Of the Vertebral Column ; Ribs*, (Figs. 25, 26 and 27.) In the Great Blue Heron the *atlas* is not large, when taken in comparison with the size attained by other vertebræ in the column, as for instance the nineteenth. Its cup for the condyle is notched above, and on either side of the neural arch above the usual blunt processes are thrown backwards (Fig. 25).

The *axis* of this bird is a very irregular bone, and a difficult one to describe without resorting to tedious detail. For this reason I have added to my illustrations a figure presenting the appearance of this bone on direct lateral view. It will be seen that that "odontoid process" is quite large, being perfectly flat above and convex below. The centrum is deep ; thinned in its centre by the laterial concavites, beneath which, its lower margin is carried by a gentle curve from the articular surface at one end to the articular surface at the other. An elongated neural crest adds another curvature to the bone above. The facets of the postzygapophyses face directly downwards, and the entire bone is much compressed from side to side. From the third to the sixth, the vertebræ are much elongated; their general pattern being seen on side view in Fig. 27. Along the median line of their neural arches above, these bones are thin and sharp. Their several articular facets are so arranged that they only permit the head to be bent forward and back again.

Fig. 6. The hyoid arches of *Ardea herodias* viewed from above. Life size from nature by the author.

The neural canal in them is small and circular on section. The 'vertebral canal' is present in all, being longest in the third vertebræ and shortest in the sixth; owing to the manner in which the parapophyses assert themselves. This is done by a foramen, which exists opposite the middle of the canal in its lateral wall; this elongates in the vertebræ from third to sixth, in a backward direction until it cuts through the hinder and outer margin of the vertebral canal of the sixth vertebra. Then a long pair of parapophyses is the result, they being very short and blunt in the third, fourth and fifth vertebræ, and only become sizable in the sixth when overtaken and developed by the advance and breaking through of the foramen in the manner indicated.

A large covered "carotid canal" is seen in the seventh to the thirteenth cervical vertebræ, inclusive; a slight deficiency taking place in the wall of the last, in the median line beneath. (*A. herodias.*) It is the most anterior part of each of these segments, and they are further characterized by being shorter and stouter than the last four described. The pneumatic foramina of these vertebræ are chiefly within the neural canal, piercing its upper wall posteriorly. From the fourteenth to the seventeenth inclusive, these vertebræ are gradually changing in form and character to resemble finally those of the dorsal region. The fifteenth is the first to show a high neural crest, with spreading diapophyses at the fore part of the vertebra, while the vertebraterial canal increases in calibre.

The neural crests or spines of the seventeenth and eighteenth are thick and long, and interlock with each other by an extensive joint.

In the *eighteenth vertebra* we observe for the first time a free pair of pleurapophyes, with very short bodies, but still articulating by tubercula and capitula.

Prof. Owen in speaking of the movement of these vertebræ of the cervical region upon one another, says: "This mechanism is most readily seen in the long-necked waders which live on fish and seize their prey by darting the bill with sudden velocity into the water. In the common Heron, for example, (*Ardea cinerea*), the head can be bent forward on the atlas or first vertebra, the first upon the second in the same direction, and so on to the sixth, between which and the fifth the forward inflection is the greatest; while in the opposite direction these vertebræ can only be brought into a straight line, From the sixth cervical vertebra to the thirteenth the neck can only be bent backward; while in the opposite direction it is also arrested at a straight line; from the fourteenth to the eighteenth the articular surface again allow of the forward inflection, but also limit the opposite motion to the straight line." ('Anat. of Verts., Vol. II, p. 39.')

This is precisely what I find in examining the same vertebræ in the neck of *Ardea herodias*. It can best be studied in the neck of a fresh specimen from which the skin has been removed, with the skeleton of the neck of another individual at hand for comparison.

The skeleton of the neck in *Nycticorax* differs in many particulars from that of *Ardea*; a number of these points only become evident after careful comparison, and will not be taken up in detail here. Others show a profound difference in organization, such as—the first pair of free pleurapophyses occurring on the seventeenth vertebra instead of on the eighteenth as in *Ardea*; the third, fourth, fifth and sixth vertebræ are not elongated as in *Ardea*, but show the simple gradation in size down the cervical chain; finally, the inferior wall of the carotid canal is open in the last four vertebræ through which it passes, in *Nycticorax*, and only in the last in *Ardea herodias*.

Returning to the nineteenth vertebra in the Great Blue Heron, we find that it has a high quadrate neural crest or spine which interlocks by a free joint with the one behind; it sends down a pair of ribs that articulate with the sternum through the intervention of a pair of costal ribs. The metapophyses are short and stumpy, barely reaching beyond the transverse processes. The bone has no descending hyapophysis, though a line marks the longitudinal centre of the centrum below. This fades away gradually on the remaining vertebræ. A large pneumatic foramen pierces the bone, on either side, behind the transverse process, and the cavities to which they lead seem to occupy all parts of the bone.

In the next four vertebræ we see but little change; they are all free elements; the neural spines do not decrease any in height, but they become shorter from before, backwards, shortest of all in the twenty-third or the *last free vertebra*, before we reach those united as one bone in the pelvis. Though this "dorsal region" the neural canal of a Heron is strikingly small, small even in proportion with the size of the vertebra. The transverse processes are narrower antero-posteriorly as we proceed backwards, but at the same time reach out further from the side of the vertebra. As we proceed towards the pelvis we note also that the facet for the head of the rib gradually approaches the anterior part of the centrum of each vertebra, but finally does not quite reach the anterior margin of the side of the neural canal in the ultimate segment. A line joins this facet in each case, with the facet for the tubercle of the rib, which is at the outer posterior angle of the diapophysis. On either side of the beam thus formed very large pneumatic openings are seen in these ultimate vertebræ, and the trabeculæ spanning the cavities within are plainly in view.

Four pairs of hæmapophyses articulate with the borders of the sterumn in all of the Herons that I have thus far examined; the fifth pair not reaching this bone, but articulating with the hinder margins of the last sternal pair. The slender pair of ribs that claim this last pair of hæmapophyses articulate with the twenty-fourth vertebra of which is the first one that anchyloses to form a part of the pelvis.

The last two pair of vertebral ribs are without epipleural appendages, and even when these processes do occur on the ribs they are very weak and freely articulated with the border. Herons have very frail ribs at the best, a fact that strikes one the moment we examine the thoracic skeleton of one of these birds.

The seventeenth vertebra having a small pair of free ribs in the Yellow-Crowned Night Heron, we find a still longer and better developed pair on the eighteenth in this form, and yet another free pair on the nineteenth. These latter have epipleural appendages, although they do not meet the sternum by costal ribs below. This gives three pairs of free ribs to *Nycticorax*; four pairs as in other Herons that meet true sternal ribs; and a pair from the pelvis, to which is attached false floating ribs, or a pair of those that articulate with the hinder borders of the preceding sternal pair proper.

*In Ardea herodias* and *A. candidissima*, the second pair of free ribs support epipleural appendages, low down on the bone.

For the moment I must now be permitted to defer our further consideration of the vertebral column, until the sternum and pectoral arch have been disposed of. After that I will return to the examination of the pelvis and coccygeal vertebræ, upon the completion of which the appendicular skeleton will finally engage our attention.

Fig. 7. The Sternum of *Ardea herodias*, viewed from below. Life size from nature.

*Of the Sternum* :—(Figs. 7, 8, 9, 31 and 32). Upon direct pectoral view, the sternum of *Ardea herodias* is seen to be broader in front than it is behind; this is due to the projection from the former end, on either side of the large costal processes, or otherwise the bone on this aspect would have a pretty regular quadrilateral figure.

The xiphoid extremity is doubly notched,—a broad triangular indentation deeply entering upon either side. This gives rise to outer xiphoidal processes, each of which point directly backwards, and have simply rounded extremities.

Evenly convex throughout, the sternal body shows but three pairs of lines upon this view—the pair of muscular lines of the pectoral muscles ; the subcostal lines ; and a pair, each one of which commences at the middle point of the inner border of the xiphoidal indentation, to be carried forwards and inwards to the carina, meeting the hinder ends of the pectoral lines.

Anteriorly, we are enabled to see the under side of the pointed manubrium, and the coracoidal beds, and gain some idea from the dissimilarity of the parts on the two sides of the former, of the method of decussation of the latter.

The anterior third of the lateral margins of the body of the sterum show, also, upon this view the little rounded elevations indicating the position of the articular facets for the hæmopophyses.

The keel fails to reach quite to the end of the sternal body behind, but is brought far up in front, commencing immediately beneath the manubrium.

Owing to the decussation of the coracoidal grooves, it depends upon which side of the sternum we view, as to how this part of the bone appears. In the drawing the right lateral view is presented, and in this particular specimen the coracoidal groove seems to have a deep triangular notch in it. Had we seen it the other way, the groove would appeared as if it ran in one continuous belt around this anterior part of the bone.

Upon this aspect, the manubrium is seen to project directly forward as a straight process. Below it, the anterior carinal margin is sharp, being concave forwards above, and straight below. The carinal angle is rounded. Muscular lines are barely seen on the side of the keel, the surface here, as it is on the sternal body above it, smooth and polished, the bone becoming only slightly thicker anteriorly below the coracoidal beds.

The keel is bounded inferiorly by an elegantly curved margin, extending from the carinal angle to nearly the end of the sternum (Fig. 8). We are now better enabled to see the hæmapophysial facets, with the deep concavities between each and its neighbor. As in so many birds, these interarticular cavities are the favorite sites of the pneumatic foramina, and they are seen to be numerous here, occupying the bottoms of the pits. For the rest of the border behind, it is sharp and continuous with the upper borde of the xiphoidal process, of which we also give a good side view.

As a whole, the costal process is triangular, with its apex at the summit of the bone.

Seen directly from above, the asymmetry of the two sides of the anterior border again becomes apparent, due to the method of articulation of the coracoids. A rounded notch exists in the median line, flanked by a long facet on the right of it, and one, only half the size on the left. The manubrium is now seen to be triangular, with its surface flat and smooth.

Well within the anterior boundary of the body of the sternum, upon this superior aspect of the bone, we observe a single elliptical foramen of some size, situate in the median line, as is its major axis. This leads to cavities in the thickened part of the front of the carina, already alluded to in a paragraph above.

From anterior border to xiphoidal extremity, and from summit of costal process to summit of costal process this sternum is one general, and by no means shallow, concavity. There are no interruptions of surface, and all these parts enter into the conformation of the basin.

For the most part it is smooth, and it is only in front that the surface seems to be roughened by some peculiar little granulations. Fig. 9, being a direct anterior view of the sternum of this Heron, the decussation of the coracoidal grooves is now best seen. The right one, (the left in the drawing) being the lower anteriorly, and running out over the top of the manubrium, while the left one, being the higher, crossing it in front.

So far as I have examined, this is the method of decussation in each instance, *i. e.*, the right hand groove being the one that passes over the superior manubrial surface.

It is just possible that this crossing of the coracoids may have arisen in the habit of the ancestors of the present Herons, of passing constantly through very narrow places, as dense cane-brakes, or such other growths of analagous character, where they probably resorted, and spent the major part of their time. There would undoubtedly be an effort made many times a day to compress the body and diminish its general bulk in a transverse direction, in such situations. Moreover, the coracoids (if arranged as in most birds) would constitute the principal obstruction to such compression; and it certainly lessens the width of the bird's body to have them crossed as they are in the Herons. If we commence sufficiently early in the life of the individual, bones and the normal position of bones may be altered very materially by gradual pressure, differently applied; then, why not, we ask, during the lapse of time, may not this result have been brought about in this way? It is hard to say, for even if it has been, then what are we to say about it, being absent in the *Rallidæ*, and present again in such forms as *Polyborus cheriway* and several other diurnal Raptores? I rarely see in any of the old-fashioned engravings, representing with the appropriate surroundings below, the noble falcon striking his prey, the doomed Heron, in mid air, that this peculiar and unique condition of the coracoids, present in both the Hawk and his quarry, does not come into my mind. Both desmognathous birds, yet evidently not related through any such physiological adaption of structure as this, arisen however it may. Still we are beginning to catch glimpses of the affinities of the *Ardeidæ* and *Falconidæ*, and morphology has much yet to bring to light in the premises. These interesting points are to be easily discovered in this anterior view of the sternum (Fig. 9). The difference in width of the hinder and anterior parts of the bone, is well shown by the relative positions of the xiphoidal and costal processes; and the thickness of the front part of the carina now becomes evident, seen from this point of view.

FIG. 8. Same bone as shown in Fig. 7, lateral view life size from nature.

The coracoids and scapulæ which I have taken the opportunity to show above, will be treated of under the head of the pectoral arch. At the lower and inner angles of the coracoids, the dotted line indicates the amount of decussation of these bones when *in situ* in their grooves on the sternum. In *A. candidissima* the sternum differs from that bone in *Ardea herodius*, as I have described it above, in only the most insignificant minor details, indeed, in all essential particulars, it is the veriest minature of the latter bone.

With *Nycticorax*, although the principal features are still there, of a Heron's sternum, yet a comparison of Figs. 8 and 32 will show that the bone has departed somewhat from the type form as seen in *Ardea*. The keel is comparatively much deeper in front and slopes up far more rapidly behind; the manubrium bears a laterally compressed plate on its anterior extremity, which is as long as the part which corresponds to the triangular portion in *Ardea*.

Finally, the main pneumatic foramen, over the keel anteriorly, is very much larger. This may contract more, however, in specimens other than the one I have in hand, and in any event is a character of very trivial importance.

## Figures and Tables

**Fig. 1. f1:**
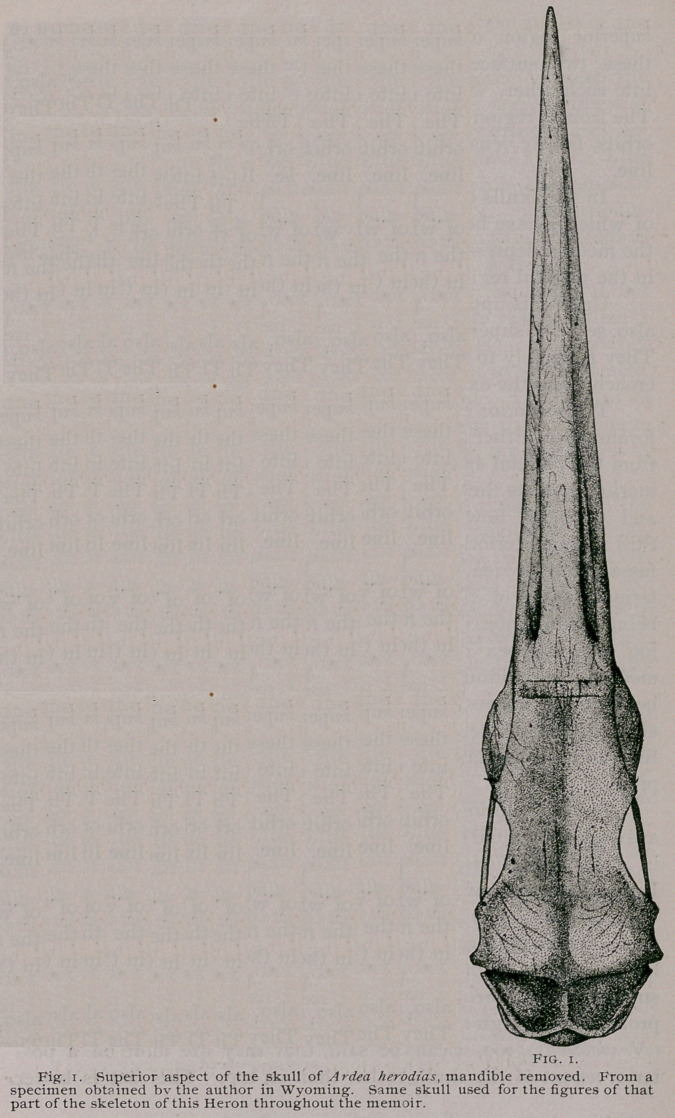


**Fig. 2. f2:**
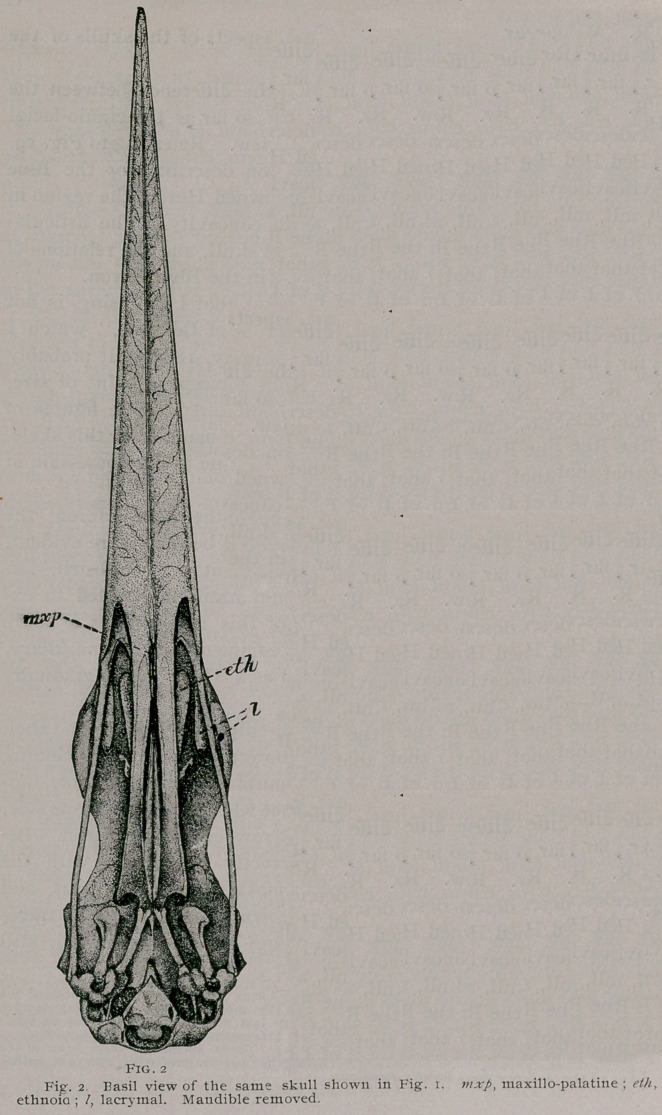


**Fig. 3. f3:**
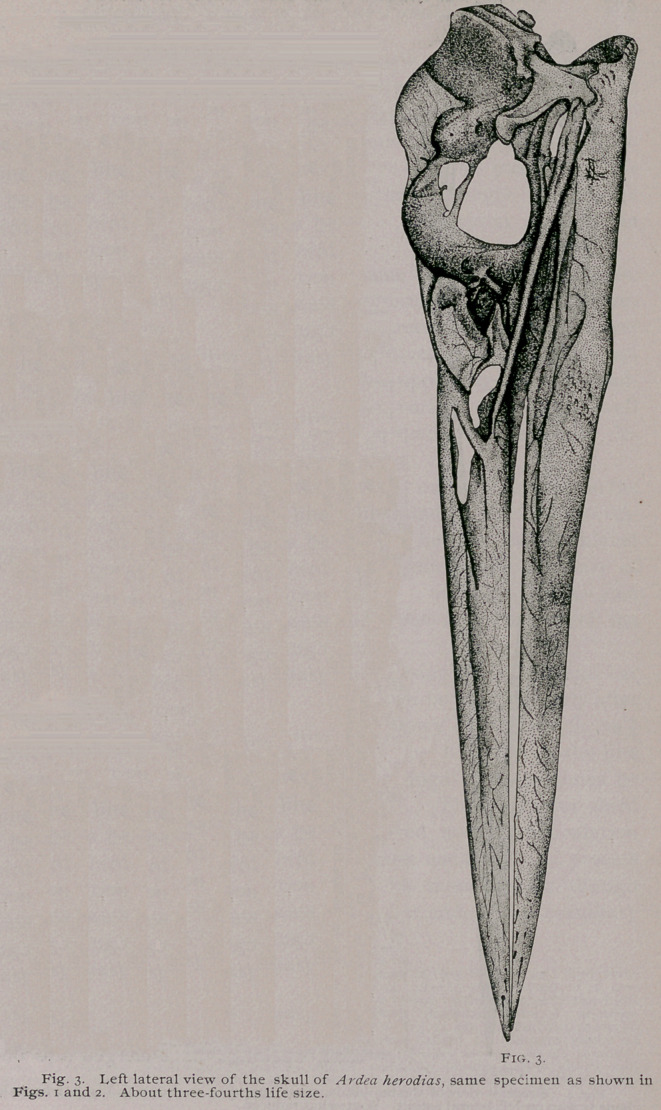


**Fig. 4. f4:**
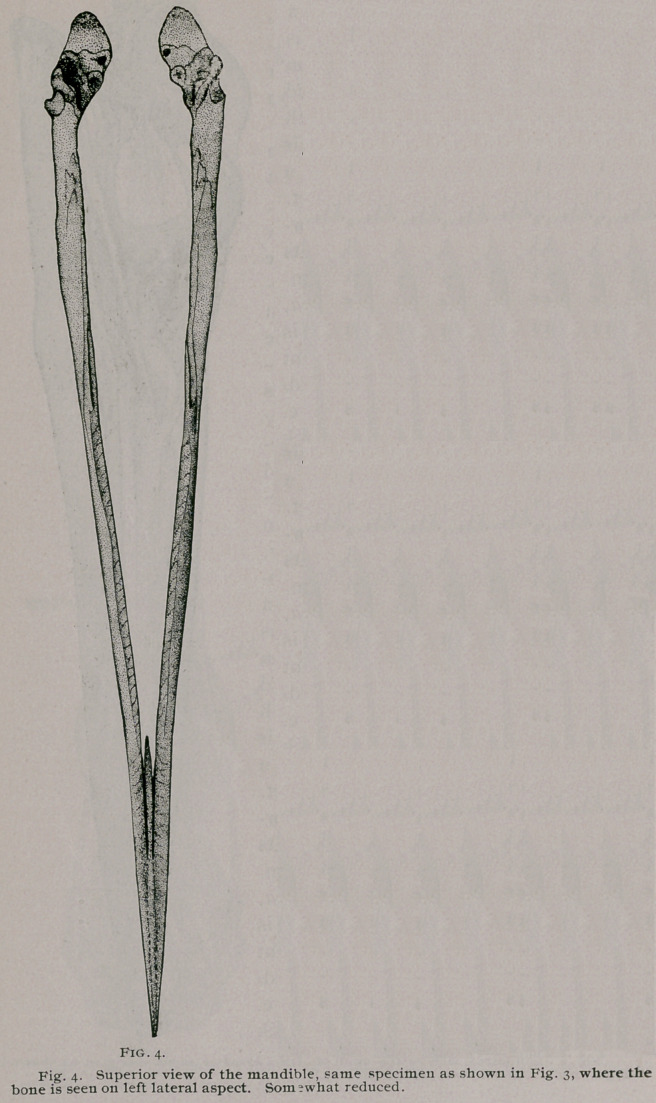


**Fig. 5. f5:**
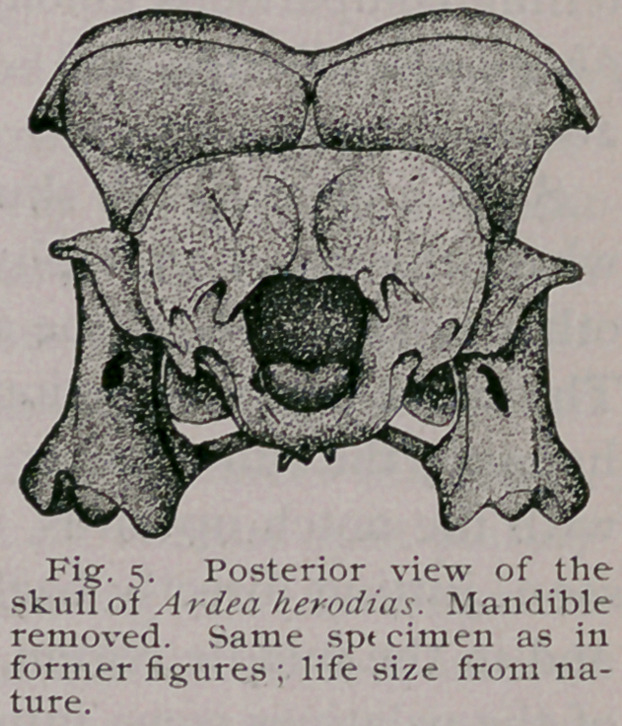


**Fig. 6. f6:**
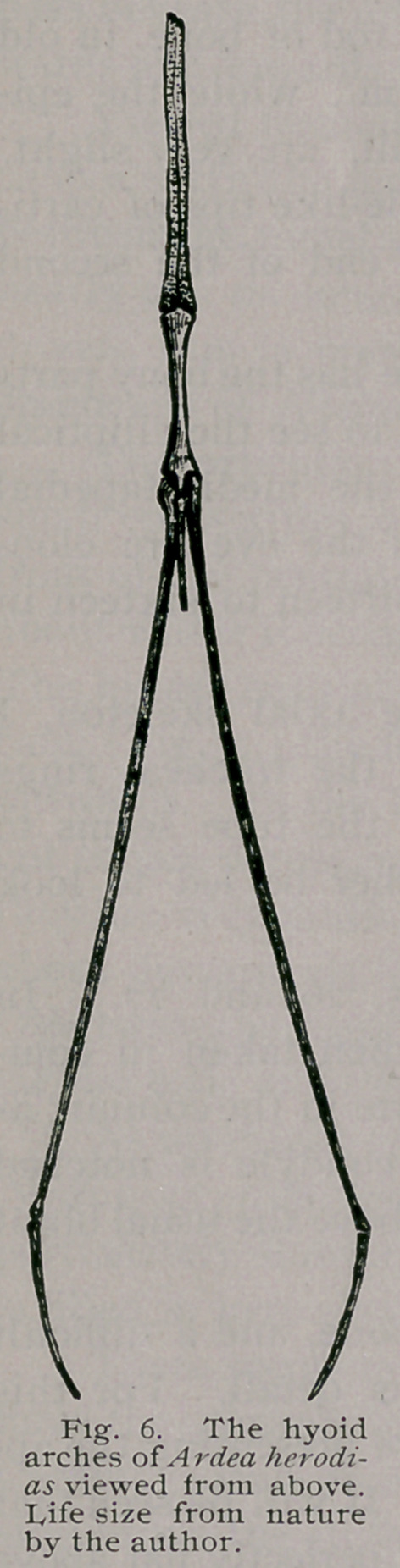


**Fig. 7. f7:**
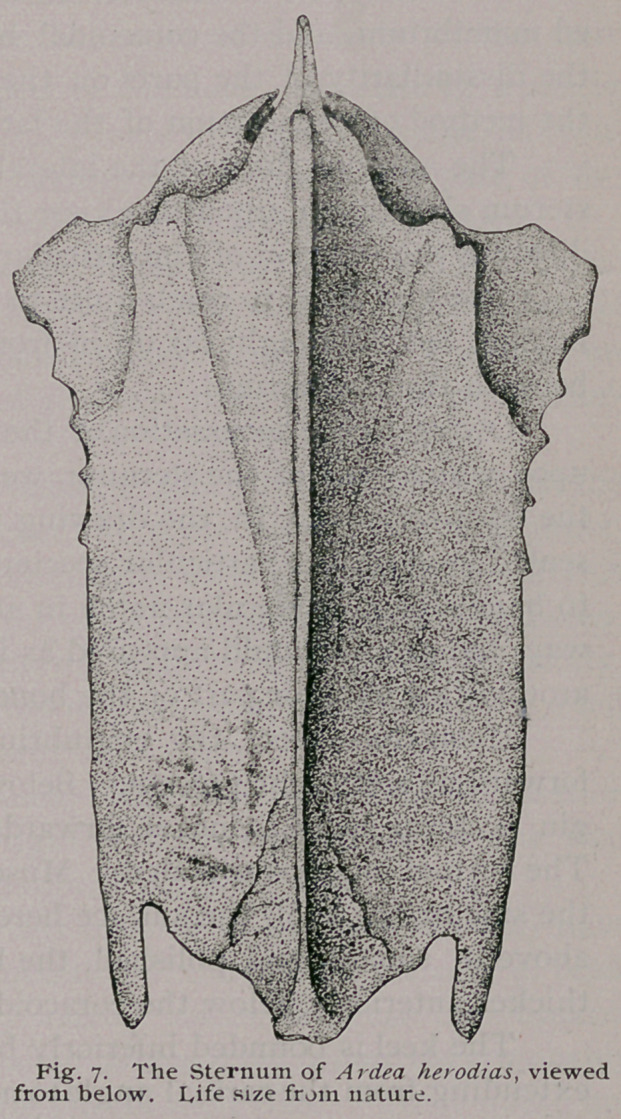


**Fig. 8. f8:**